# Development of Sustainable Construction Materials from Inert Waste Mixtures Using the Mechanosynthesis Process

**DOI:** 10.3390/ma17174301

**Published:** 2024-08-30

**Authors:** Rabah Hamzaoui, Othmane Bouchenafa, Rachida Idir, Assia Djerbi, Teddy Fen-Chong, Céline Florence, François Boutin

**Affiliations:** 1 Institut de Recherche, ESTP/Université Paris-Est, 28 Avenue du Président Wilson, 94234 Cachan, France; cflorence@estp.fr; 2 Microbusiness (Low Carbon Construction Materials), 29 Avenue Leon Blum, 94230 Cachan, France; 3 Concrete4change Ltd., Unit 4-5-7—The Heathcoat Building, Nottingham Science & Technology Park, University Boulevard, Nottingham NG7 2QJ, UK; o.bouchenafa@concrete4change.com; 4 Cerema, Univ Gustave Eiffel, UMR MCD, F-77171 Sourdun, France Cerema, 110 Rue de Paris, 77171 Sourdun, France; rachida.idir@cerema.fr; 5 Univ Gustave Eiffel, Cerema, UMR MCD, 5 Boulevard Descartes Champs-sur-Marne, 77454 Marne-la-Vallée Cedex, France; assia.djerbi@univ-eiffel.fr (A.D.); teddy.fen-chong@univ-eiffel.fr (T.F.-C.); 6 CSTB, 84 Avenue Jean Jaurès, Champs-sur-Marne, 77454 Marne-la-Vallée Cedex, France; francois.boutin@cstb.fr

**Keywords:** mechanosynthesis, inert waste, cement formulations, reactivity enhancement, sustainable construction materials

## Abstract

This research investigates the potential of mechanosynthesis to transform inert waste mixtures into sustainable construction materials. Three waste streams were employed: recycled glass, recycled concrete, and excavated soils. Two alternative material formulations, F1 (50% recycled concrete, 30% recycled glass, 20% excavated soil) and F2 (60% excavated soil, 20% recycled concrete, 20% recycled glass), were developed. Cement pastes were produced by partially substituting cement (CEM I) with 50% of either F1 or F2. Characterization techniques, including X-ray diffraction (XRD), Fourier transform infrared spectroscopy (ATR-FTIR), and mechanical testing, were performed. Cement pastes incorporating milled waste materials exhibited significantly enhanced compressive strength compared to their unmilled counterparts. At 28 curing days, compressive strengths reached 44, 47, 45, and 49.7 MPa, and at 90 curing days, they increased to 47.5, 50, 55, and 61 MPa for milling conditions of 200 rpm for 5 min, 200 rpm for 15 min, 400 rpm for 5 min, and 400 rpm for 15 min, respectively. In addition, F1 formulations showed higher compressive strengths than the reference CEM II and CEM III pastes. These results highlight the efficacy of mechanosynthesis in valorizing construction waste, mitigating CO_2_ emissions, and creating environmentally friendly construction materials.

## 1. Introduction

Globally, construction and demolition activities generate an immense volume of waste, exceeding 10 billion tons annually [1]. A significant portion, ranging from 60% to 80% depending on countries and world regions, falls under the classification of inert waste [2,3,4,5]. In France, 260 million tons of construction and demolition waste are generated yearly, with more than 70% considered inert waste. The recycling rate for inert construction and demolition waste ranges from 48% to 64%, involving transforming this waste into valuable products like construction aggregates or backfill materials [6,7,8]. 

The disposal of inert waste is becoming more regulated with the aim of reaching a 100% recycling rate. As per European Directive 1999/31/EC [9], inert waste can be defined as “waste that does not undergo significant physical or chemical changes, does not biologically degrade, and does not produce harmful leachate in the environment”. This category includes mineral waste from construction and demolition activities, such as concrete, bricks, tiles, glass, excavated soil, debris, pebbles, industrial by-products like slag, fly ash, silica fume, and other items like shells.

Cement consumption is continually increasing due to human needs. This consumption is predicted to increase from 4.13 Gt/year to 4.68 Gt/year between 2016 and 2050, respectively [10,11]. Cement production is accompanied by a large amount of global CO_2_ emissions into the atmosphere. Each ton of clinker produced is responsible for 0.8 tons of CO_2_ released into the atmosphere. Therefore, for several years, many studies have focused on reducing the rate of CO_2_ emitted by the cement industry in order to achieve the objectives, which consist of limiting global warming to 1.5 °C and achieving zero greenhouse gas emissions by 2050 [12].

This study aims to answer these two issues discussed above, “waste management and CO_2_ reduction”, by creating new cement mix designs with good performances depending on the desired application while replacing part of the clinker with other constituents of natural or industrial origins. Many materials are used to substitute clinker in binary and ternary cement mix designs, such as fly ash, slags, limestone, metakaolin and silica fume. Some types of these binary and ternary cements have already been studied, and their specifications (composition and use) are standardized according to the standard EN 197-1 [13], such as CEM II, III, IV and V. Others are being evaluated by the NF EN 197-5 [14], as a new type of cement, CEM II-C-M, that consists of a rate of substitution of clinker between 36 and 50% by a mixture of several constituents. The CEM VI is also under evaluation in this standard [14] based on a high rate of substitution: 51–65% of clinker by a slag content between 31 and 59%, to which other constituents are also added. New standard EN 197-6 [15] cement based on fine recycled concrete with a substitution reach of 35% has been utilized since 2023. 

In addition to these standards, several researchers have investigated different ways for incorporating waste materials, such as excavated soil, recycled glass, and recycled concrete, into cement formulations for the development of low-carbon cements and concrete [16,17,18,19,20]. 

The use of recycled glass in cementitious materials is particularly promising. For example, Idir et al. [16] demonstrated that incorporating glass fines into mortars, with up to 40% cement (CEM I) substitution, could inhibit alkali-silica reaction (ASR) while improving mechanical properties. Research shows that glass fines have pozzolanic properties, contributing to the formation of calcium-silicate-hydrate (C-S-H) and enhancing the durability and strength of concrete. Moreover, the combined use of coarse glass aggregates and glass fines can reduce mortar expansion due to ASR while maximizing the use of recycled glass. Sadiqul Islam et al. [17] observed that mortars and concretes incorporating milled glass and replacing 20% of the cement exhibited enhanced compressive strength compared to control samples. This substitution also offers a potential reduction in production costs and a decrease in CO_2_ emissions by 18%. Given that waste glass is non-biodegradable and typically ends up in landfills, utilizing it in concrete production not only mitigates environmental impact but also significantly improves the material’s mechanical and durability characteristics. These benefits are well-documented across multiple studies [16,17,18], underscoring the optimal use of glass in concrete applications. 

The use of excavated soils in cementitious materials offers significant potential for the valorization of construction waste. These soils from construction sites and infrastructure projects are often considered inert waste but possess interesting properties for the fabrication of cementitious materials. Studies have shown that excavated soils can be effectively used in traditional cement formulations as a replacement for cement or sand [19], and, moreover, in alkali-activated matrices [21]. 

The valorization of recycled concrete fines presents a challenge due to their low intrinsic reactivity. These fines, resulting from demolishing and recycling concrete, are often considered inert waste, limiting their use in traditional cement formulations. Various activation methods have been explored to overcome this limitation, especially grinding methods [6,20]. Bola Oliveira et al. [20] employed a planetary ball mill (Retsch-PM100, Schönwalde-Glien, Germany) at 500 rpm for milling times of 0.5, 2, and 6 h, replacing 7%, 15%, and 25% of Portland cement with milled recycled concrete (RCP). They reported that cement formulations containing 15% and 25% RCP met the C40 and C32 class requirements, respectively, as per Brazilian technical standards. This substitution has the potential to reduce CO_2_ emissions by up to 25%. Bouchenafa et al. [6], utilized mechanosynthesis for activating recycled concrete fines. This process involves intensive mechanical grinding of the recycled concrete powders, increasing their reactivity. They achieved significant results by substituting 50% of CEM I with activated recycled concrete powders (FRC) in mortars. The compressive strengths were considerably enhanced compared to those using non-activated FRC, with increases of +110%, +61%, and +68% after 1, 7, and 28 days of curing, respectively. Although these performance metrics are lower than those of a mortar composed entirely of CEM I, they still meet the mechanical standards of CEM II and CEM III cements. These results highlight the potential of mechanosynthesis for better utilization of recycled materials in cement formulations, offering a promising solution to reduce CO_2_ emissions from clinker-based cements and valorize construction waste.

Mechanosynthesis is a promising technique for fabricating advanced materials through intensive mechanical grinding of precursor powders. This high-energy process induces remarkable transformations, including the formation of metastable alloys inaccessible by traditional solidification methods and the production of nanostructured materials via the breakdown of microcrystals into nanocrystals. This approach also enables the synthesis of various inorganic compounds such as oxides, nitrides, and carbides [22,23,24,25,26]. 

Mechanosynthesis uniquely facilitates the preparation of novel materials, such as alloys and advanced composites, by combining different elements at room temperature. For example, a nanostructured bcc Fe-Ni solid solution was produced from iron and nickel mixtures by Hamzaoui et al. [26]. Similarly, CaCu_3_Ti_4_O_12_ electroceramics were synthesized through high-energy ball milling of CaO, CuO, and TiO_2_ powders by Dullan et al. [27]. 

Although ball milling is commonly employed in civil engineering to activate materials through grinding, its potential for creating new compounds from mixed powders, as demonstrated in ceramics and metallurgy, is largely untapped in this field. 

This observation is pivotal to the EMADIM project (*Elaboration de Matériaux Alternatifs à partir de Déchets Inertes par Mécanosynthèse*/Development of Alternative Materials from Inert Waste through Mechanosynthesis), which is aimed at developing alternative construction materials from inert waste using mechanosynthesis. Innovative methods to valorize waste by combining different materials and applying mechanosynthesis to enhance their properties are being explored in this project. New materials are proposed to be created by ball milling various inert wastes, including glass, recycled concrete, excavated soil, shells, and roof tiles. Mechanosynthesis is employed as a mechanical activation process, modifying particle size and shape while preserving the material structure at short milling times. However, significant changes in the crystallographic structure can be induced by extended milling, leading to the formation of new compounds. 

This study focuses on developing an innovative construction material by replacing 50% of CEM I cement with a mechanochemically activated mixture of recycled glass, recycled concrete, and excavated soil. These wastes were selected due to their potential for valorization, their inherent properties, and the urgent need for sustainable waste management strategies. This approach not only valorizes waste streams but also reduces the carbon footprint of cement production, offering dual environmental benefits.

## 2. Materials and Methods

### 2.1. Materials

**Figure 1:**
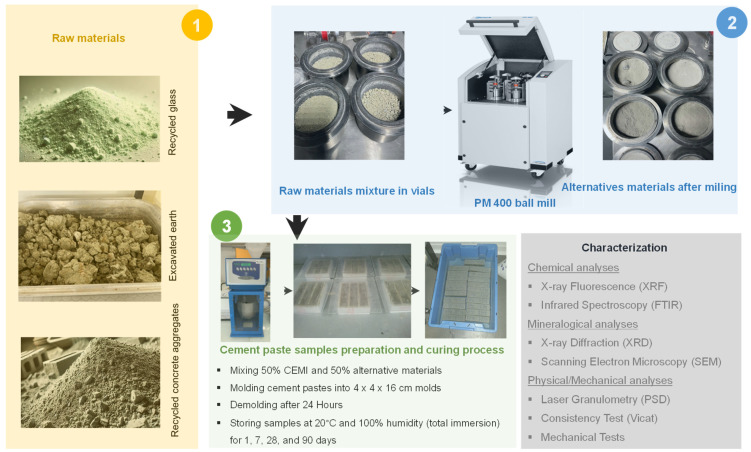
Schematic representation of the steps from raw materials to the preparation of alternative materials and the manufacturing of cement pastes and modified cement paste samples.

The cement used in this study is a CEM I 52.5 N CE CP2 NF, with a clinker content ranging between 95 and 100%. This cement complies with the European standard NF EN 197-1. It was used in the mix designs instead of raw clinker due to its high clinker content. CEM II (B-L) 32.5 from Calcia, France and CEM III/C 32.5 from Calcia, France have been used for mechanical test comparisons. The recycled glass was sourced from the Paris region, France and crushed in the laboratory. Recycled concrete aggregates were obtained from northern France, and the excavated earth was taken from the large construction project “Grand Paris” and crushed in the laboratory (see Figure 1). The chemical composition is summarized in Table 1.

### 2.2. Methods

#### 2.2.1. Alternative Material Powder Preparation

Currently, the cement industry uses three main indicators to adjust the raw meal composition based on their process: lime saturation factor (LSF), silica modulus ratio (SM), and alumina modulus ratio (AM) [27].

(1) $$LSF=\frac{CaO}{2.8{SiO}_{2}+1.2{Al}_{2}O_{3}+0.65{Fe}_{2}O_{3}}$$

(2) $$SM=\frac{{SiO}_{2}}{{Al}_{2}O_{3}+{Fe}_{2}O_{3}}$$

(3) $$AM=\frac{{Al}_{2}O_{3}}{{Fe}_{2}O_{3}}$$

Following this approach, two indicators have been proposed for selecting the optimal blending of inert wastes and adapting alternative materials in this study:

(4) $$PZ_{h}=\frac{CaO}{SiO_{2}+Al_{2}O_{3}}$$

(5) $$HD_{h}=\frac{CaO}{SiO_{2}+Al_{2}O_{3}+Fe_{2}O_{3}}$$

PZ_h_ (4): This equation is an indicator that can help assess the pozzolanic reactivity of formulation. Pozzolanic reactivity refers to the ability of a material (like clay) to react with lime (CaO) and form cementitious compounds such as C-S-H. The presence of silicates and aluminates in the formulation is key for this reactivity.

HD_h_ (5): This equation provides information on the formulation’s hydraulic reactivity. Hydraulic reactivity indicates how much lime (CaO) is consumed by other ingredients to form hydrates, which are essential for the material’s setting and hardening.

PZ_h_ and HD_h_ range from the interval 0.1 to 0.6 and consider the hydraulic and pozzolanic properties of various calcined clays (containing lime) found in the literature and used for cement substitution. In addition to the previously mentioned factors, the selection of formulations also considers the combined effect of the different crystallographic phases.

The two formulations presented in this work are F1 (50% recycled concrete + 30% recycled glass + 20% excavated earth) and F2 (60% excavated earth + 20% recycled concrete + 20% recycled glass). Table 2 summarizes the chemical composition and indicators of the two selected recomposed binders.

Once the formulations were selected, the recomposed final product was milled using a Retsch PM 400 planetary high-energy ball mill (Figure 1). This mill consists of four vials mounted on a rotating disc. As the disc rotates, the vials move in a circular path opposite to the direction of the disc’s rotation. Two disc rotation speeds were selected: 400 rpm and 200 rpm. For each speed, the milling times were 5 and 15 min. The weight of the powder sample in each vial was 260 g. After one milling stage, a total weight of 1 kg was obtained. The ball-to-powder weight ratio was 3.1. The selected grinding conditions, specifically rotation speed and grinding time, were determined based on previous studies, both published [6,28] and unpublished.

According to the technical datasheet provided by RETSCH, the PM400 grinder consumes a total power of 2.2 kWh at its maximum rotation speed of 400 rpm. Table 3 presents a detailed calculation of the power consumption for various grinding conditions.

#### 2.2.2. Sample Paste Preparation

According to the NF EN 196-3 standard [29], the amount of water necessary to form a cement paste is determined using the manual Vicat test. The Vicat device was used to determine the water/cement ratio that ensured good hydration and workability (standardized consistency) for each formulation. The reference cement paste was prepared by mixing cement (C) and water (W) with a W/C ratio of 0.33. Modified cement pastes with alternative materials (F1 or F2) substituted at 50% by weight were prepared with a W/(C + F1) ratio of 0.33 and a W/(C + F2) ratio of 0.45.

Figure 1 summarizes the different steps from raw materials to the manufacturing of cement paste samples. In the first step, raw materials are prepared in granular form with a maximum particle size of 10 mm. Subsequently, the raw material mixture is meticulously distributed into vials to ensure consistent doses for each grinding batch. Grinding is then performed using the PM400 under specified conditions. The resulting ground powder (alternative material) is collected in bags. In the third step, cement pastes composed of 50% CEM I and 50% alternative materials are prepared in molds measuring 4 × 4 × 16 cm^3^. After 24 h, the pastes are demolded and cured in 100% immersion in water at 20 °C for 7, 28, and 90 days. Finally, both the ground powders and the cured pastes will undergo characterization using appropriate techniques. 

#### 2.2.3. Characterization Techniques

The chemical composition was analyzed by X-ray fluorescence spectrometry (XRF) using a Bruker S2 Ranger instrument.

Mineralogy was determined by X-ray diffraction (XRD) using a Bruker D2 Phaser diffractometer equipped with a Cu-Kα X-ray tube (λ = 1.54 Å). Samples were scanned over an angular range of 5° to 60° (2θ) with a step size of 0.02° and a dwell time of 0.2 s. Data analysis was performed using DIFFRAC.EVA™ software (https://www.bruker.com/en/products-and-solutions/diffractometers-and-x-ray-microscopes/x-ray-diffractometers/diffrac-suite-software/diffrac-eva.html accessed on 3 July 2024) with the International Centre for Diffraction Data (ICDD) PDF4 database (PDF: Powder Diffraction File).

The particle size distribution was determined using a laser particle analyzer (LS 13 320 XR, Beckman Coulter, Brea, CA, USA). A liquid module known as the Universal Liquid Module (ULM) was utilized with ethanol as the dispersing solvent to prevent particle agglomeration. The PGP/alcohol ratio was maintained at 1:20 during the analysis, and an ultrasonic generator was employed throughout the process.

Infrared (IR) spectroscopy measurements were conducted using a PerkinElmer Spectrum Two instrument with attenuated total reflectance Fourier transform infrared (ATR-FTIR) spectrometry. Spectra were collected in the 4000–400 cm^−1^ range with a resolution of 4 cm^−1^ and 64 added scans.

Scanning electron microscopy observations were conducted using a Gemini microscope model SUPRA 55VP (Zeiss, Oberkochen, Germany) coupled with an energy dispersive spectroscopy (EDS) probe.

Compressive and flexural strengths of the manufactured cement and modified cement pastes (see Figure 1) were assessed using a 3R (Syntech, Toulouse, France) 300 kN machine at different curing ages: 1, 7, 28, and 90 days. For each processing condition, three samples with dimensions 4 × 4 × 16 cm^3^ were tested for flexural strength and six for compressive strength, following the EN 196-1 standard [30].

## 3. Results and Discussion

### 3.1. Characterization of Raw Materials and Alternative Material Powders

**Figure 2:**
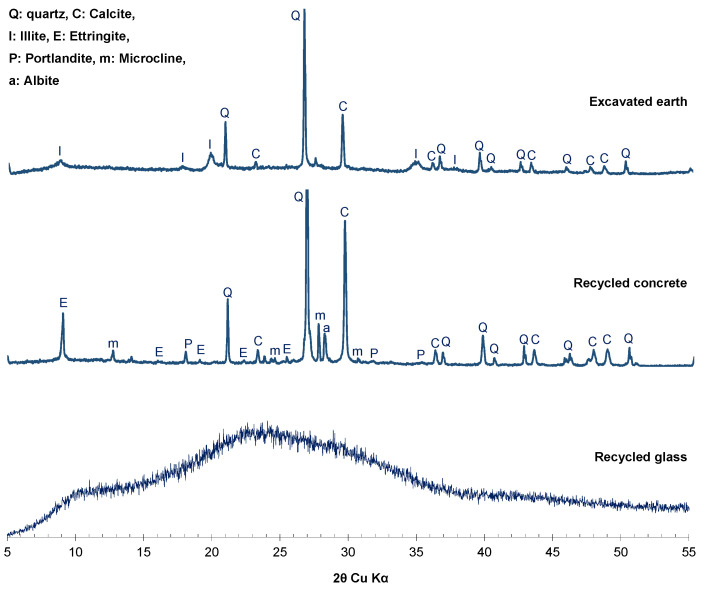
XRD patterns of raw materials: recycled glass, recycled concrete and excavated earth.

The coarse fragments of the raw materials (recycled glass, recycled concrete and excavated soils) were crushed using a Retsch BB50 jaw crusher from Germany and sieved to a particle size of 125 μm. The resulting powder was then analyzed using X-ray diffraction (XRD), and the results are presented in Figure 2.

The diffraction pattern of the recycled glass showed a broad hump (halo) observed between 20° and 40° (2θ), indicative of an amorphous phase.

For the recycled concrete, the following crystallographic phases were observed: quartz (PDF 00-003-0444), calcite (PDF 04-002-9082), ettringite (PDF 04-027-9475), portlandite (PDF 00-044-1481), albite (PDF 01-071-1150), and microcline (PDF 00-019-0926). The literature has reported these same phases [6,31].

In the case of the excavated soils, the identified phases included quartz (PDF 00-003-0444), calcite (PDF 04-002-9082), albite (PDF 01-071-1150), and illite (PDF 00-002-0050), classifying this earth as illitic clay. Quartz and calcite are major phases in both recycled concrete and illitic clay.

**Figure 3:**
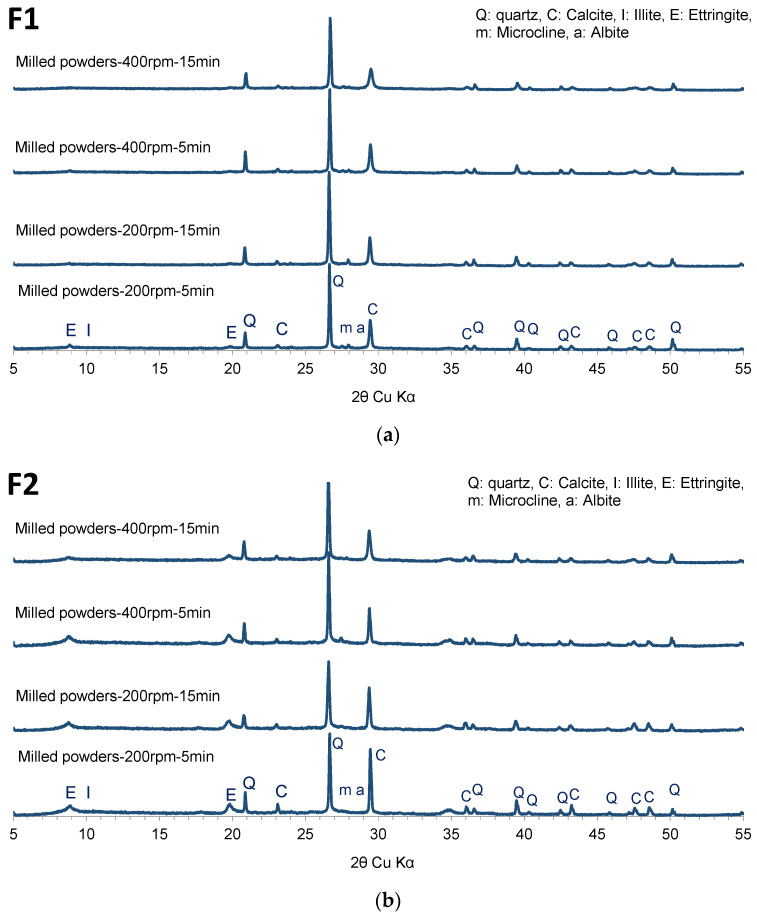
XRD patterns of (**a**) recomposed materials F1 at 200 rpm for 5 min, 200 rpm for 15 min, 400 rpm for 5 min, 400 rpm for 15 min; (**b**) recomposed materials F2 at 200 rpm for 5 min, 200 rpm for 15 min, 400 rpm for 5 min, 400 rpm for 15 min.

Figure 3a,b present the diffraction patterns of the recomposed materials F1 and F2, respectively. The patterns correspond to milling conditions of 200 rpm for 5 and 15 min and 400 rpm for 5 and 15 min.

A significant decrease in peak intensities was observed in both materials (F1 and F2), suggesting the total amorphization of portlandite and partial amorphization of ettringite, microcline, and albite. However, the XRD pattern for F1 milled processed at 400 rpm for 15 min showed a complete disappearance of peaks, indicating the total amorphization of all the mentioned phases. Using a planetary ball mill for the mechanical activation of recycled concrete, Bouchenafa et al. [6] observed the complete amorphization of portlandite and ettringite phases after only 5 min of milling. The authors attributed this amorphization to the accumulation of internal stresses within the crystalline material caused by plastic deformations induced by ball milling. These stresses can manifest as point defects, dislocations, and other imperfections in the crystal lattice. Additionally, a near-total amorphization of the illite phase was observed in the F1 formulation under milling conditions of 200 rpm for 15 min, 400 rpm for 5 min, and 400 rpm for 15 min. Conversely, the illite phase was always present in the F2 formulation under all milling conditions, although some peak disappearances were noted.

For the major phases in both formulations (F1 and F2), quartz and calcite, a broadening of peaks and a decrease in intensity were observed compared to the raw materials (recycled concrete and excavated earth). This effect was more pronounced under high-energy milling conditions of 400 rpm for 15 min. This can be attributed to the increased energy input during milling at 400 rpm. As the rotation speed increases, the milling energy increases, leading to a higher degree of internal stress within the crystal lattice. Generally, the peak broadening and intensity decrease observed in the mechanosynthesis process are attributed to the formation of nanostructured or nanocrystalline materials [28].

Using ball milling for a mixture of limestone and clay, Hamzaoui and Bouchenafa [28] observed the narrowing of the peaks for quartz and calcite in the X-ray diffraction (XRD) pattern as a function of milling time. This can be attributed to the reduction in crystallite size and nanostructured quartz and calcite formation. They calculated the crystallite size of both minerals and found that it decreased from 29.2 ± 1.1 nm to 17.2 ± 1.1 nm after 60 min of milling for quartz and from 22 ± 1.1 nm to 8.9 ± 1.1 nm for calcite. The authors explained that this decrease was caused by the mechanical forces introduced during milling, which generated stresses in the powder by generating dislocations and other defects. These defects promoted a reduction in grain size, generally due to the reduction in diffusion distances.

**Figure 4:**
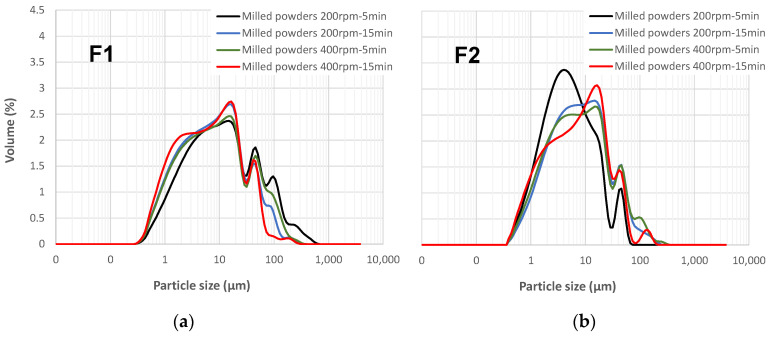
Particle size distribution analysis of F1 and F2 under different milling conditions (200 rpm for 5 and 15 min, 400 rpm for 5 and 15 min) (**a**): F1 (**b**): F2.

Laser diffraction and scanning electron microscopy (SEM) are commonly used techniques to study the granularity and morphology of different powders. For F1 and F2, milled under various conditions (200 rpm for 5 min, 200 rpm for 15 min, 400 rpm for 5 min, and 400 rpm for 15 min), the particle size distribution (PSD) is shown in Figure 4a,b, respectively.

The particle size distribution (PSD) for F1 milled at 200 rpm for 15 min, 400 rpm for 5 min, and 400 rpm for 15 min exhibited a bimodal distribution. However, the PSD for F1 milled at 200 rpm for 5 min displayed a trimodal distribution. This distribution had two peaks similar to the bimodal ones but also included a third peak between 92 µm and 170 µm with a maximum at 110 µm. This rightward shift of the PSD for the 200 rpm-5 min condition and the emergence of the third peak suggested agglomeration due to the less intense milling compared to the other conditions. Table 4 summarizes the particle sizes (D_10_, D_50_, and D_90_) of F1 and F2 under various milling conditions (200 rpm for 5 and 15 min, 400 rpm for 5 and 15 min). It is evident from the table that the D(10), D(50), and D(90) values for F1 milled at 200 rpm for 5 min were higher compared to the other F1 formulations, suggesting larger particle sizes due to agglomeration during the less intense milling process.

The particle size distribution (PSD) of F2 milled under various conditions is presented in Figure 4b. For material F2 milled at 200 rpm for 5 min, the PSD exhibited a bimodal distribution with two distinct peaks. The first peak fell between 0.3 µm and 30 µm, with a maximum of around 3.9 µm. The second peak lay between 30 µm and 82 µm, reaching a maximum at approximately 45 µm. In contrast, F2 milled at 200 rpm for 15 min and 400 rpm for 5 min displayed similar PSDs. These distributions were also bimodal, with the first peak range matching that of the 200 rpm 5 min condition (maximum around 10.7 µm) and sharing the same second peak range. However, F2 milled at 400 rpm for 15 min presented a trimodal distribution. While the first two peak ranges mirrored those previously observed, an additional, smaller third peak emerged between 80 µm and 145 µm, with a maximum around 115 µm.

A comparison of F1 and F2 reveals a clear difference in their agglomeration behavior. F1 exhibited agglomeration primarily under low-intensity milling conditions, whereas F2 showed minimal agglomeration until subjected to more intense milling conditions (200 rpm for 15 min or higher). This difference can be attributed to the higher clay content in F2, as clay is typically characterized by fine particles that are more prone to agglomeration under high-energy milling than the materials present in F1.

The impact of the mechanosynthesis process on particle behavior can be divided into three stages [22,23,28]:

- Rearrangement and Stacking: Particles slide past each other with minimal deformation or fracturing, leading to a decrease in particle size and a change in shape.
- Elastic and Plastic Deformation: Particles experience both elastic and plastic deformations, which are often characterized by “cold welding”, where particles stick together, causing an increase in particle size through agglomeration.
- Fracture: Particles are broken apart, resulting in smaller particles and potentially additional fragmentation, leading to a significant reduction in particle size.

The extent of agglomeration depends on the material being processed. For some materials, like those prone to cold welding, even mild milling conditions (low speed and short time) can lead to agglomeration. In contrast, agglomeration might occur for materials that readily fracture, like clays, and then be broken apart during subsequent milling cycles.

**Figure 5:**
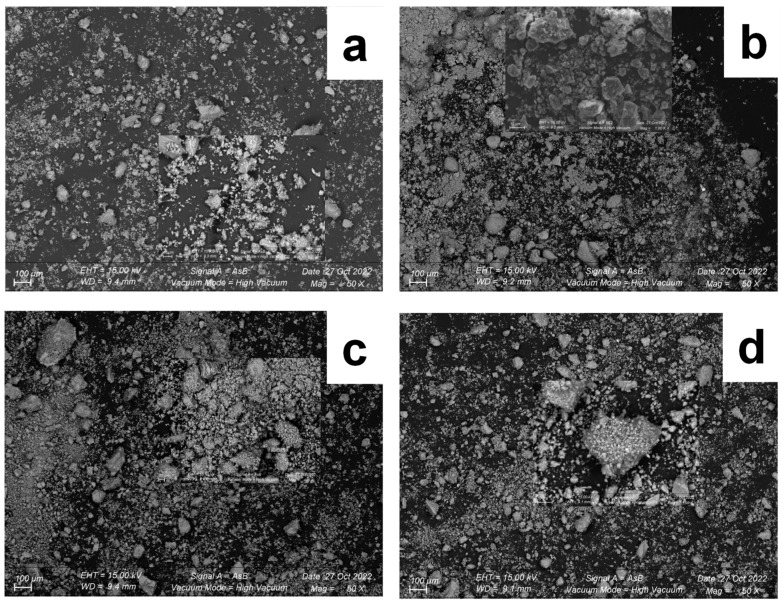
SEM images of F1 and F2 under different milling conditions (200 rpm for 15 min and 400 rpm for 15 min) (**a**): F1, 200 rpm for 15 min, (**b**): F1, 400 rpm for 15 min, (**c**): F2, 200 rpm for 15 min, (**d**): F2, 400 rpm for 15 min.

Figure 5 displays micrographs of formulations F1 and F2 obtained under milling conditions of 200 rpm for 15 min and 400 rpm for 15 min. For formulation F1, the micrographs reveal fine particles with various shapes, including angular, spherical, and irregularly shaped particles of various sizes. This observation is consistent with the particle size distribution (PSD) analysis, which shows the presence of fine particles after 15 min of milling at different rotation speeds.

The SEM image of F2 shows that the particles primarily exist as stacks and flat, irregularly shaped structures. Additionally, particles of various sizes are evident, contributing to the observed distributions in Figure 4. Furthermore, the phenomenon of agglomeration of thin particles is prevalent, resulting in the presence of opaque zones. In mechanosynthesis, particle size reduction, shape changes, and particle agglomeration are common phenomena arising from the interplay of fracturing and welding forces during the milling process. These forces lead to particle impacts and entrapment between grinding balls or between the balls and the vial walls [22,23].

### 3.2. Characterization of Cement Paste and Modified Cement Pastes

**Figure 6:**
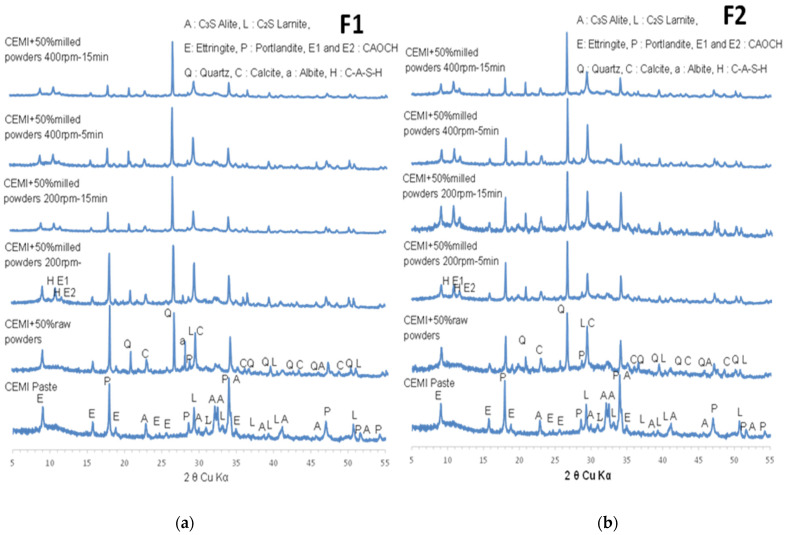
Diffraction patterns of CEMI cement paste and F1 (**a**) and F2 (**b**) cement pastes for unmilled raw materials and under different milling conditions (200 rpm for 5 min, 200 rpm for 15 min, 400 rpm for 5 min, and 400 rpm for 15 min).

To use unmilled raw materials (a mixture of recycled glass, recycled concrete and excavated soil) as a reference for modified cement pastes, F1 and F2 were crushed using a Retsch BB50 jaw crusher and sieved to a particle size of 125 μm. Figure 6a,b present the diffraction patterns of the CEM I cement paste, and cement pastes F1 and F2 for both unmilled raw materials and different milling conditions (200 rpm for 5 min, 200 rpm for 15 min, 400 rpm for 5 min, and 400 rpm for 15 min).

For the CEM I cement paste, the presence of various phases such as Portlandite (PDF-00-004-0733), Ettringite (PDF-04-013-3691), Alite (C_3_S, PDF-00-042-0551), and Larnite (C_2_S, PDF-00-033-0302) was observed. These phases are characteristic of CEM I pastes and have been reported by several researchers in the literature [6,31].

In the cement pastes with unmilled raw materials F1 and F2, all the phases identified in the CEM I paste were found, along with quartz (PDF-00-003-0444) and calcite (PDF-04-002-9082), which are the principal phases of the alternative materials.

The XRD analysis indicated that milling increased the amorphization of the raw materials, reducing the intensity of the crystalline peaks. Higher milling speeds and longer milling times resulted in more significant amorphization, with near-complete amorphization observed at 400 rpm for 15 min.

Formulations F1 and F2 followed a similar trend, with increasing milling intensity leading to higher amorphization. Formulations F1 (left) and F2 (right) show comparable results under similar milling conditions, but F2 may have slightly more residual crystalline phases under certain conditions, possibly due to its different initial composition. Regarding the cement pastes with milled materials (F1 and F2), it was shown that in addition to all phases found in ordinary Portland cement (OPC) paste and cement paste with unmilled F1 and F2, there are two peaks that can be attributed to the formation of C-A-S-H by the Cowlesite phase (PDF-00-029-0286). This phase has been reported in the literature related to high silicate concentration used in geopolymers and cement hydration [32]. Its presence here can be justified by the high silica content of materials (especially from glass) and the intensive grinding process. These conditions can mimic the environments in which Cowlesite forms, even outside of traditional geopolymer systems. These two peaks can also be attributed to the identified calcium aluminum oxide carbonate hydrate (CAOCH) based on reference patterns (PDF-00-036-0129, left peak; PDF-00-014-0083, right peak). A more in-depth study will be conducted using nuclear magnetic resonance (NMR) spectroscopy and scanning electron microscopy (SEM) for pastes to distinguish between these different phases.

The changes observed in the XRD patterns are consistent with the typical effects of mechanosynthesis, which induces structural modifications and reduces crystallinity through mechanical activation. These observations suggest that the mechanosynthesis process effectively alters the crystalline structure of the materials, potentially increasing their reactivity and suitability for use in alternative cement formulation.

**Figure 7:**
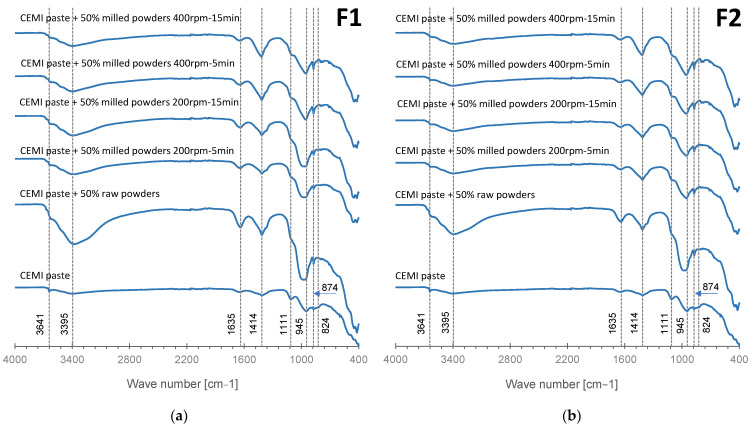
Infrared spectra of CEM I cement pastes, (**a**) F1 cement pastes, and (**b**) F2 cement pastes.

Figure 7 presents the infrared spectra of CEM I cement pastes, (a) F1 cement paste, and (b) F2 cement paste. For the IR spectra of modified cement pastes with unmilled alternative materials, both F1 and F2 show bands similar to those observed in the CEM I cement paste. However, some modifications in peak shape are present, such as rounded peaks for portlandite (3641 cm^−1^) and C-S-H (945 cm^−1^). These changes suggest that the formation of these phases is negatively affected by the addition of unmilled alternative materials. Similar behavior was observed for F2 formulations prepared at 200 rpm for 5 and 15 min, with a C-S-H peak appearing at 824 cm^−1^. Interestingly, F2 formulations were prepared at 400 rpm for 5 and 15 min, and all F1 formulations exhibited bands similar to those of the CEM I cement paste with high peak intensity. Notably, C-S-H peaks at 945 cm^−1^ and 824 cm^−1^ were well-defined, especially in samples prepared under high milling conditions (400 rpm for 15 min). These observations suggest that high milling conditions promoted reactivity in both F1 and F2 formulations, leading to broader bands that potentially indicate enhanced hydration and pozzolanic reactions.

These modifications may improve the overall reactivity of the materials, making them promising candidates for the valorization of inert waste in cement formulations.

Lastly, the high intensity of the bands around 1414 cm^−1^ and 874 cm^−1^, corresponding to carbonate group vibrations, can be attributed to the presence of carbonates in the recycled concrete and excavated earth used in the formulations.

**Figure 8:**
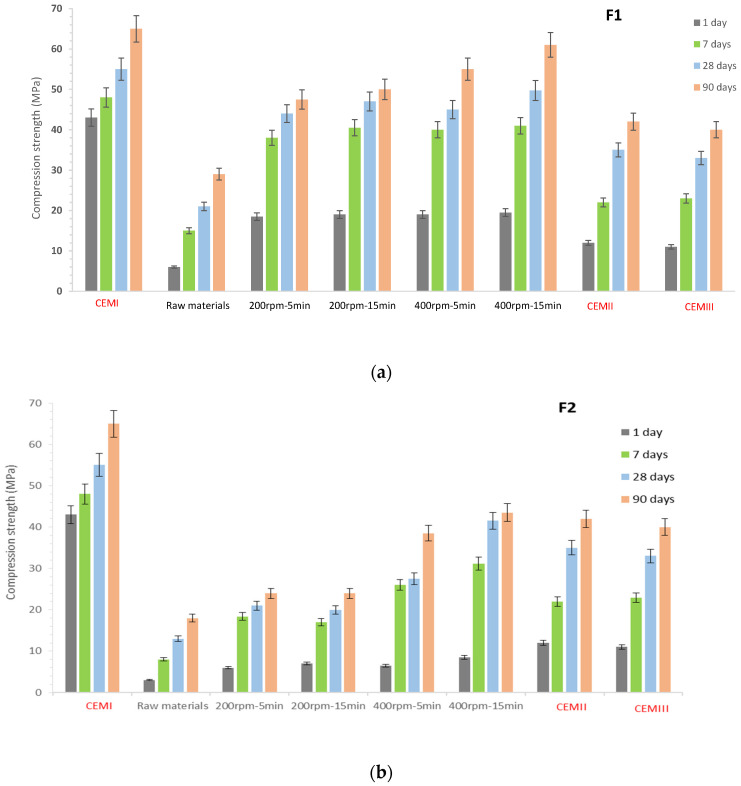
The evolution of compressive strength of reference cement pastes (CEM I, CEM II, and CEM III) and cement pastes with F1 (**a**) and F2 (**b**).

The results of compression strength tests on cement pastes and modified cement pastes with F1 and F2 (50% CEM I substitution) are presented in Figure 8a,b.

The reference cement paste prepared with CEM I (without substitution) exhibited compressive strengths of 43, 48, 55, and 65 MPa after 1, 7, 28, and 90 days of curing, respectively. For the CEM II reference cement paste, the compressive strength reached 12, 22, 35, and 42 MPa, and for the CEM III reference cement paste, it reached 11, 23, 33, and 40 MPa after 1, 7, 28, and 90 days of curing, respectively. For modified cement pastes, unmilled F1 and F2 exhibited lower compressive strengths than CEM II and III pastes. These formulations reached strengths of 6, 15, 21, and 29 MPa (F1) and 3, 8, 13, and 18 MPa (F2) after 1, 7, 28, and 90 curing days, respectively. This indicates a lack of reactivity and interaction between the components (recycled concrete, excavated earth, and recycled glass) in the unmilled state.

Cement pastes with milled F1 (Figure 8a) formulations exhibited higher compressive strengths than the reference CEM II and CEM III pastes. Compressive strength reached 44, 47, 45, and 49.7 MPa at 28 curing days and 47.5, 50, 55, and 61 MPa at 90 curing days under different milling conditions of 200 rpm for 5 min, 200 rpm for 15 min, 400 rpm for 5 min, and 400 rpm for 15 min, respectively.

The strength increase in the F1 formulation can be attributed to the high amorphization rate of the raw materials after milling, the nanostructure of dominant phases (quartz and calcite), and the increased presence of fine particles. Mechanosynthesis not only improves particle dispersion but also increases their specific surface area, thus promoting a better pozzolanic reaction contributing to C-S-H formation.

**Figure 9:**
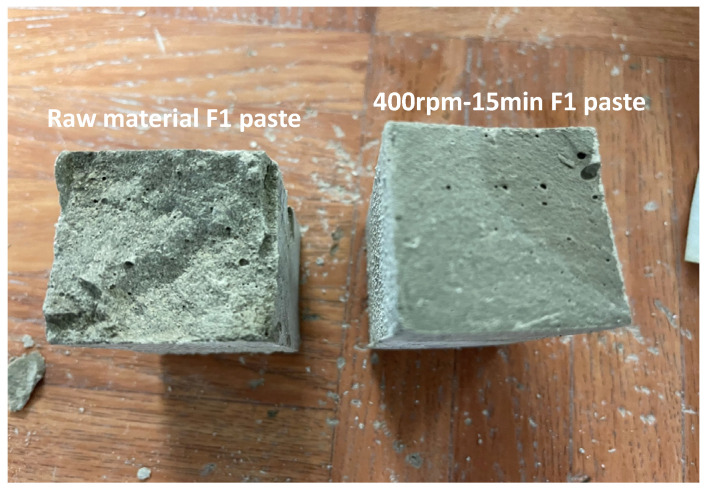
Surface state of unmilled F1 paste and F1 paste milled at 400 rpm for 15 min after mechanical test at 28 curing days.

Comparing the state and shape of samples (after compressive strength testing) of the unmilled F1 formulation and F1 milled at 400 rpm for 15 min (see Figure 9), a granular surface was observed for the unmilled F1 paste and a smooth surface for the milled sample. This smooth plane is attributed to the brittle behavior due to the significant presence of C-S-H gel in the sample, which provides high resistance.

Concerning milled F2, it also demonstrated superior performance compared to the unmilled version, with two formulations (400 rpm, 5 min and 15 min) achieving compressive strengths close to those of the CEM II and III reference pastes. For the 400 rpm, 5-minute formulation, compressive strengths reached 27.5 and 38.7 MPa at 28 and 90 curing days, respectively. For the 400 rpm, 15-minute formulation, compressive strengths reached 41.3 and 43.7 MPa at 28 and 90 curing days, respectively.

The strength increase in these two formulations can be attributed to plastic deformations occurring at 400 rpm (maximum speed rotation) and the formation of nanostructured material, as evidenced by XRD peaks of calcite, which were broader and of lower intensity compared to others, confirming the formation of nanocrystalline calcite. Generally speaking, high-energy ball milling involves several parameters affecting the properties of the final products, including milling speed, milling time, and the ball-to-powder ratio. Recent studies have shown that milling speed directly impacts the final product and can be compensated for by milling time, requiring the selection of longer milling durations [33].

Comparing formulations F1 and F2, it was observed that F1 is more suitable than formulation F2 for cement replacement regardless of the milling conditions used. These results confirm the potential of mechanosynthesis to valorize inert waste into high-performance and durable cement formulations.

In this section, the carbon footprint of the formulations has been calculated. The CO_2_ emissions, measured in kilograms of CO_2_ equivalent per ton (Kg CO_2_ eq/t) of raw material produced using a planetary mill, were determined based on straightforward calculations, focusing specifically on the carbon balance rather than a full life cycle assessment (LCA). This evaluation excluded emissions related to the transport of materials, thus concentrating solely on the direct impacts of the grinding process. The calculations, as presented in Table 3, used data from the French electricity grid, which were sourced from a platform providing real-time information on electricity production and CO_2_ emissions in France (30 g CO_2_ eq/kWh) [34]. It is important to note that from the grinding process, 1 kg of alternative material was yielded. Following these simplified yet focused conversions and calculations, Table 5 presents the results in Kg CO_2_ eq/ton for alternative materials under various milling conditions.

The main conclusions from the analysis of CO_2_ equivalent emissions based on varying milling times and speeds include several observations. Shorter milling times clearly showed that reduced grinding durations result in lower CO_2_ equivalent emissions, suggesting that minimizing the time spent grinding can significantly reduce the environmental footprint of the production process. Regarding the impact of increased speeds, although higher speeds tend to increase emissions, the increase is not exponential unless the grinding is prolonged. Therefore, higher speeds could be used effectively if they are maintained for shorter durations, thus balancing the need for efficient material processing with environmental considerations.

**Figure 10:**
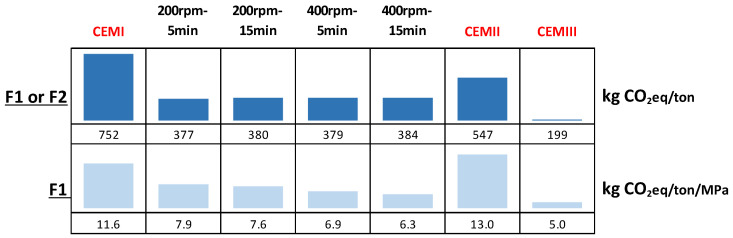
Comparative analysis of CO_2_ impact for alternative and conventional cements under different grinding conditions.

Figure 10 illustrates the CO_2_ equivalent impact for the production of 1 ton of alternative materials (F1 or F2) under various grinding conditions, compared to that of conventional cements (CEMI, CEMII, and CEMIII), with the CO_2_ impacts for the conventional cements sourced from French production data [35]. It also presents the CO_2_ impact per megapascal (MPa) of compressive strength achieved at 90 days of curing for each material.

It can be observed from Figure 10 that the CO_2_ impact of producing the different formulations, regardless of grinding speed and duration, is lower than that of CEM I and CEM II cements. The emissions ranged from 377 kg CO_2_eq/ton for the lowest value (200 rpm for 5 min) to 384 kg CO_2_eq/ton for the highest (400 rpm for 15 min).

Regarding CEM III, with its 199 kg CO_2_eq/ton, this cement proved to be the least impacting in terms of CO_2_ emissions. This performance is attributable to its composition, which incorporates more than 86% slag, a product with reduced environmental impact. However, given the uncertainties concerning the future availability of slag, it becomes crucial to explore other, more readily available and less environmentally impactful options.

Figure 10 also offers a comparative analysis of the CO_2_ impact per megapascal (MPa) of compressive strength, expressed in kg CO_2_eq/ton/MPa. This approach provides a more comprehensive perspective, allowing for the evaluation of environmental efficiency relative to the mechanical performance of the materials. The gap in terms of CO_2_ impact narrows between the different alternative materials and CEM III, thanks to better resistance achieved with these alternative materials. Although the impact of CEM I is significantly reduced, it remains higher than that of the alternative materials.

## 4. Conclusions

This study explored the use of mechanosynthesis to valorize inert waste in cement formulations. The main findings are as follows:

1. Material property enhancements:
  - Intensive ball milling (400 rpm, 15 min) significantly amorphized alternative materials, enhancing their reactivity.
  - Nanostructuring of quartz and calcite was observed in F1 and F2 formulations after milling.
  - Milled materials demonstrated improved compressive strength compared to their unmilled counterparts.
2. Compressive strength performance:
  - The modified F1 formulation surpassed reference CEM I and CEM III cements in compressive strength tests at 28 and 90 days, achieving particularly high strengths.
  - For F1, milling at 400 rpm for 15 min achieved a high compressive strength of 61 MPa at 90 days.
  - Additionally, the F1 formulation outperformed the F2 formulation in cement replacement, regardless of milling conditions.
3. Impact on CO_2_ emissions:
  - Reducing milling time correlated with lower CO_2_ equivalent emissions, indicating a positive correlation between milling duration and environmental impact.
  - Compared to CEM I and CEM II cements, all formulations exhibited reduced CO_2_ impacts, with decreases of 49.8% and 29.5%, respectively.
4. Strategy for construction waste management:
  - Incorporating inert waste (recycled glass, recycled concrete, excavated earth) into cement formulations suggests a promising strategy to reduce the cement industry’s carbon footprint while addressing construction waste management effectively.

## Figures and Tables

**Table 1:** Chemical composition of raw materials.

Samples/Elements (%)	CaO	SiO_2_	Al_2_O_3_	Fe_2_O_3_	MgO	K_2_O	TiO_2_	SO_3_	Na_2_O
Recycled glass	14.24	66.62	1.86	0.11	2.10	0,70	0.29	0.31	13.80
Recycled concrete	42.34	42.23	7.30	3.90	1.85	1.80	0.50	1.50	0.93
Excavated soils	13.68	53.27	15.92	8.23	4.12	5.02	1.10	0.69	0.24

**Table 2:** Chemical composition and indicators (PZ_h_ and HD_h_) of recomposed binders F1 and F2.

Samples/Elements (%)	CaO	SiO_2_	Al_2_O_3_	Fe_2_O_3_	MgO	K_2_O	TiO_2_	SO_3_	Na_2_O	PZ_h_	HD_h_
F1	24.25	52.93	7.47	2.70	2.70	2.00	0.47	0.79	6.1	0.40	0.38
F2	18.57	52.37	13.12	5.83	3.50	3.72	0.86	0.50	2.6	0.28	0.26

**Table 3:** Power consumption (W) for various grinding conditions.

Milling Time (mm)/Milling Rotation Speed (rpm)	200	400
5	91.67 W	183.33 W
15	275.00 W	550.00 W

**Table 4:** Particle sizes (D10, D50, and D90) of F1 and F2 under various milling conditions (200 rpm for 5 and 15 min, 400 rpm for 5 and 15 min).

Samples/Particles Size	D_10_ [µm]	D_50_ [µm]	D_90_ [µm]
F1 200 rpm-5 min	1.66	11.16	77.48
F1 200 rpm-15 min	1.24	8.38	44.69
F1 400 rpm-5 min	1.27	8.50	58.58
F1 400 rpm-15 min	1.09	7.33	39.72
F2 200 rpm-5 min	1.24	4.94	20.82
F2 200 rpm-15 min	1.19	4.72	17.22
F2 400 rpm-5 min	1.29	8.50	58.58
F2 400 rpm-15 min	1.18	8.52	36.13

**Table 5:** CO_2_ equivalent emissions per ton of alternative materials produced using a planetary ball mill under various milling conditions.

Milling Conditions	200 rpm-5 min	200 rpm-15 min	400 rpm-5 min	400 rpm-15 min
KgCO_2_equi/t	2.75	8.25	5.50	16.50

## Data Availability

The raw data supporting the conclusions of this article will be made available by the authors on request.
